# Expression of Sex Hormone Receptor and Immune Response Genes in Peripheral Blood Mononuclear Cells During the Menstrual Cycle

**DOI:** 10.3389/fendo.2021.721813

**Published:** 2021-09-22

**Authors:** Peik M. A. Brundin, Britt-Marie Landgren, Peter Fjällström, Mohamed M. Shamekh, Jan-Åke Gustafsson, Anders F. Johansson, Ivan Nalvarte

**Affiliations:** ^1^Department of Clinical Microbiology, Infection and Immunology, and the Laboratory for Molecular Infection Medicine Sweden, Umeå University, Umeå, Sweden; ^2^Department of Biosciences and Nutrition, Karolinska Institutet, Huddinge, Sweden; ^3^Unit of Infectious Diseases, Department of Medicine, St Göran’s Hospital, Stockholm, Sweden; ^4^Kvinnohälsan, Karolinska University Hospital, Huddinge, Sweden; ^5^Department of Biochemistry, Faculty of Veterinary Medicine, Assiut University, Assiut, Egypt; ^6^Center for Nuclear Receptors and Cell Signaling, University of Houston, Houston, TX, United States

**Keywords:** menstrual cycle, estrogen receptor, progesterone, sex hormone, immune response, estrogen

## Abstract

Sex hormones are known to interact with the immune system on multiple levels but information on the types of sex hormone receptors (SHR) and their expression levels in immune cells is scarce. Estrogen, testosterone and progesterone are all considered to interact with the immune system through their respective cell receptors (ERα and ERβ including the splice variant ERβ2, AR and PGR). In this study expression levels of SHR genes in peripheral blood mononuclear cells (PBMCs) and cell subsets (CD4^+^ and CD8^+^ T-cells, CD56^+^ NK-cells, CD14^+^ monocytes and CD19^+^ B-cells) were analyzed using standard manual qPCR or a qPCR array (TLDA). Nine healthy individuals including men (*n* = 2), premenopausal (Pre-MP, *n* = 5) and postmenopausal (post-MP, *n* = 2) women were sampled for PBMCs which were separated to cell subsets using FACS. Ten Pre-MP women were longitudinally sampled for total PBMCs at different phases of the menstrual cycle. We found that ERα was most abundant and, unexpectedly, that ERβ2 was the dominant ERβ variant in several FACS sorted cell subsets. In total PBMCs, SHR (ERα, ERβ1, ERβ2, and AR) expression did not fluctuate according to the phase of the menstrual cycle and PGR was not expressed. However, several immune response genes (*GATA3, IFNG, IL1B, LTA, NFKB1, PDCD1, STAT3, STAT5A, TBX21, TGFB1, TNFA*) were more expressed during the ovulatory and mid-luteal phases. Sex hormone levels did not correlate significantly with gene expression of SHR or immune response genes, but sex hormone-binding globulin (SHBG), a steroid hormone transporting protein, was positively correlated to expression of ERβ1 gene. This study provides new insights in the distribution of ERs in immune cells. Furthermore, expression patterns of several immune response genes differ significantly between phases of the menstrual cycle, supporting a role for sex hormones in the immune response.

## Introduction

Men and women are affected differently by infectious diseases, with higher male mortality and morbidity from infectious diseases (1). One major reason to this may be that the immune response differs between men and women (2). In general, women mount a stronger response than men towards pathogens and/or seem to clear the pathogen more effectively (2). Men are more prone to contract certain infectious diseases related to differences in behavior (3), but even when controlling for exposure, women seem to have a benefit (4). For specific infectious diseases (e.g., severe dengue fever) a strong immune response could be detrimental, and therefore be a disadvantage for women (3).

A growing body of evidence suggests that sex hormones may both augment and dampen the immune response (5). The female advantage in mortality to infectious diseases decreases from the 5^th^ decade of life (1). As this coincides with the female menopause and decreasing levels of female sex hormones, it is plausible that estradiol (E_2_) and progesterone (P_4_) have roles in shaping the immune response (1, 2). Furthermore, autoimmune diseases are more common in women, a phenomenon also partly attributed to sex hormones (6).

The menstrual cycle involves fluctuation of P_4_ and E_2_ levels as well as follicle-stimulating hormone (FSH) and luteinizing hormone (LH). FSH stimulates the ovarian follicles to produce E_2_, which is necessary for the mid-cycle sharp surge of LH that initiates the ovulation. If fertilization does not occur, the corpus luteum breaks down and P_4_ levels drop. As the levels of hormones shift, so might the immune response, affecting the temporal severity of autoimmune and infectious diseases (7, 8).

Sex hormone receptors (SHRs) have been found in several non-reproductive tissues, and sex hormones may affect e.g., bone density, muscular growth and blood coagulation. Not surprisingly, sex hormone receptors have also been found in several types of immune cells (9). SHRs include estrogen receptor (ER)α, ERβ, androgen receptor (AR) and progesterone receptor (PGR), and belong to the steroid activated nuclear receptor family of transcription factors (10). These receptors are intracellular and may upon ligand stimulation bind directly to DNA sequences, or tether with transcription factors (e.g., NFκB, AP-1 and SP1) to mediate gene transcription of among other immune related genes, such as type I interferons (IFN-α and IFN-β) (5). The two subtypes ERα and ERβ are found in several splice variants (isoforms), the latter including ERβ1-5, of which several have been associated to disease development by e.g., antagonizing full-length ERα or ERβ (10–12).

In the present study, we sampled pre-menopausal (pre-MP) women over four phases of the menstrual cycle to analyze variation in serum hormone levels, expression of SHRs, and several key immune response genes in peripheral blood mononuclear cells (PBMCs). Our data demonstrate that the expression of several immune response genes changes over the menstrual cycle and that the ERβ splice variant, ERβ2 that cannot bind E2, may be more prominent in this process than full length ERβ (ERβ1). This study adds new evidence to the sex differences in immune response.

## Materials and Methods

### Participants

Healthy premenopausal women (pre-MP, *n* = 15), postmenopausal women (post-MP, *n* = 2) and men (*n* = 2) were included according to a protocol approved by the Central Ethical Review Board (Swedish Research Council, Stockholm, Dnr: Ö 24–2009), and body-mass index (BMI) and age were registered for all subjects. For women, parity, menstrual cycle length or years since initiation of menopause was noted. Exclusion criteria were (1) medication with hormonal replacement therapy, or contraceptives during the last three months (2), Regular medication with ASA, NSAIDs (e.g., ibuprofen and diclofenac), morphine, morphine-derivatives or cortisone compounds (3), pregnancy or childbirth within the last year and (4) irregular or perimenopausal bleeding.

### Sampling Procedure

All the samples were collected at 8–10 a.m. The samples from menstruating females were collected during one menstrual cycle, early in the follicular phase (cycle day 1–3), during mid-follicular phase (day 8–10), and at the ovulatory phase day 12-15. The follicular size was measured by ultrasound. The day of the LH and FSH peaks was determined by using Ovustick (Monoclonal Antibodies, Mountain View, CA, USA) in urine from day 12 until the day after the LH peak and in the luteal phase (5–7 days after the day of the LH-Peak and FSH peak). Ovulation was confirmed when progesterone levels were above 22 nmol/mL. Similarly, the post-MP females and male participants were sampled once a week on the same weekday over four weeks. At every time-point, serum analysis was performed for hormones (S-estradiol, S-testosterone, S-progesterone, S-prolactin, S-FSH, S-LH, S-SHBG, S-TSH, S-T4) and blood cells complete blood count and differential count (lymphocytes, monocytes, neutrophils, basophils and eosinophils). Vacutainer CPT mononuclear cell preparation tubes (BD Biosciences, Franklin Lakes, NJ, USA) were used according to the manufacturer’s description to separate PBMCs from whole blood. PBMCs were slowly frozen in 20% DMSO and Heparin solution and kept at -135°C. Serum samples were drawn, left to coagulate at room temperature for 30 min, and then centrifuged for 10 min at maximum speed before storing at -20°C. The blood samples were drawn at Kvinnohälsan (Karolinska University Hospital, Huddinge) and analyzed at the Karolinska University Laboratory (KUL, Huddinge, Sweden) and analyzed as previously described (13). Separate serum samples were also drawn to estimate 5-α dihydrotestosterone, (performed at HUS-lab, Helsinki, Finland), using a liquid chromatography-tandem mass spectrometry method (LC-MS/MS).

### Fluorescence-Activated Cell Sorting

Frozen PBMC samples from pre-MP (*n* = 5), post-MP (*n* = 2) and males (*n* = 2), collected at 4 different time-points (as described above) were prepared for cell storing using FACS. The samples were thawed in a 37°C water bath and diluted with ice-cold PBS followed by 2 washing steps with ice-cold PBS by centrifugation (300 x g, 5 min) at 4°C. The cell pellet was resuspended in 200 μL ice-cold PBS. The cell suspension was incubated in darkness with respective antibodies (CD3 PE-Cy 7, Cat No 341111; CD4 PerCP-Cy 5.5, Cat No 332772; CD8 APC-H7 RUO, Cat No 641400; CD56 PE (MY31), Cat No 345810; CD19 APC (SJ25C1), Cat No 345791, all from BD Biosciences (San Jose, CA, USA), and CD14 [DakoAgilent, Santa Clara, CA, USA)], for 15 minutes and diluted with 2 mL PBS before centrifugation (600 x g, 5 min) at 4°C. Unbound antibodies (supernatant) were discarded and the cell pellet was resuspended in 400 μL PBS followed by cell sorting using FACSAria (BD Biosciences). At least 30’000 cells were collected from each category before storing at -80°C.

### RNA Extraction and cDNA Synthesis

RNA was extracted using the Qiagen RNeasy kit (Qiagen, Hilden, Germany) and cDNA synthesis performed using High-Capacity cDNA Reverse Transcription Kit (Thermo Fisher Scientific, Vilnius, Lithuania) with random hexamers, according to manufacturer’s instructions.

### Manual qPCR on Sorted Cells and PBMCs

cDNA from both unsorted and FACS sorted PBMCs (CD4^+^ T-cells, CD8^+^ T-cells, CD56^+^ NK-cells, CD14^+^ monocytes and CD19^+^ B-cells) were used analyzed by qPCR using 0.5 µl cDNA, 300 nM forward and reverse primers (ERα: forward, 5’-GAATCTGCCAAGGAGACTCGC -3’; reverse, 5’-ACTGGTTGGTGGCTGGACAC-3’; ERβ1, forward, 5’- TCCATGCGCCTGGCTAAC -3’; reverse, 5’- CAGATGTTCCATGCCCTTGTTA -3’; ERβ2, forward, 5’- TCCATGCG

CCTGGCTAAC -3’; reverse, 5’- CCATCGTTGCTTCAGGCAA -3’; GR forward 5’-GAGCAGTGGAAGGACAGCA-3’; reverse,

5’-TTTCTTCGAATTTTATCGATGATGC-3’; GPER1, forward, 5’- TCACGGGCCACATTGTCAAC; reverse 5’- GTCTCCCCGAGAAAGCTGTAG-3’; and GAPDH: forward, 5’-CCCATCACCATCTTCCAG-3’; reverse, 5’-ATGACCTTGCCCACAGCC-3’), and SYBR green FAST PCR master mix according to manufacturer’s instructions (Applied Biosystems, Foster City CA, USA). The qPCRs were setup and run on a 7500 FAST real-time PCR system (Applied Biosystems, Foster City, CA, USA) and relative mRNA expression was analyzed using the ΔCt method relative to GAPDH expression.

### Taqman Low Density PCR-Array Analysis

PBMC cDNA from Pre-MP women (*n* = 10) with complete set of samples representative for different phases of the menstrual cycle was mixed with TaqMan Fast Advanced master mix (Applied Biosystems) and RNase-free water. cDNA mix was loaded into each of the 8 loading ports of a Taqman low density Array (TLDA, Applied Biosystems). The array was sealed, centrifuged 2 minutes at 1800 x g, and the following qPCR performed on a 7900HT qPCR system (Applied Biosystems) with ABI software SDS v2.4 installed using standard TLDA array cycling. GAPDH was used as reference gene for ΔCT calculations using the ABI software RQmgr 1.2.1 followed by DataAssist v3.0 (Applied Biosystems). Each sample was analyzed in triplicates for each of the 30 genes assayed, including sex hormone receptors, proinflammatory markers as well as T_H_1-, T_H_2-, Treg- and T_H_17-related immunological markers (**Supplemental Table 1**). The qPCR results are presented as ΔCT values to allow linear model analyses on normal distributed values.

### Statistical Analyses

Student’s t-test with Welch’s correction was used to compare the amount of ERα, ERβ1 and ERβ2 in the different PBMC cell subsets. Linear mixed modeling (LMM) was used to estimate the effect of sampling timepoints and gene expression similarly as described by us before (14). In brief, the R-package *nlme* was used for LMM analysis where timepoint was tested as fixed effect, and the expression of the various genes for each pre-MP individual was set as random effects, and *p*-values were calculated. Bonferroni correction was used to adjust the significance level of *p*-values relative to the number of repeated LMMs for the different genes studied. The repeated measures correlation test (15) was used to determine associations between gene-gene expressions and between gene expression and hormone levels.

## Results

### Clinical Characteristics

Brief characteristics of 19 unique individuals that donated blood samples for analysis by manual qPCR on FACS-sorted cells or a Taqman low density PCR array (TLDA) on total PBMCs are shown in **Table 1**.

**Table 1:** Clinical characteristics of participants, range (median).

Analysis	Subject group	Age	Parity	Menstrual cycle length in days	Years since last menses	BMI
qPCR on FACS-sorted cells and PBMCs	Pre-MP (*n* = 5)	25–32 (31)	0–2 (0)	28–31 (28)	–	21.5–27.5 (22.0)
	Post-MP (*n* = 2)	60 and 61	0 and 2	–	10 and 14	25 and 23.7
	Males (*n* = 2)	21 and 68	–	–	–	21.9 and 23.8
Taqman PCR array on total PBMCs	Pre-MP (*n* = 10)	24–36 (31,5)	0–2 (0)	21–31 (28)	–	17.9–27.5 (22.4)

### Distribution of ERs in PBMCs

**Figure 1:**
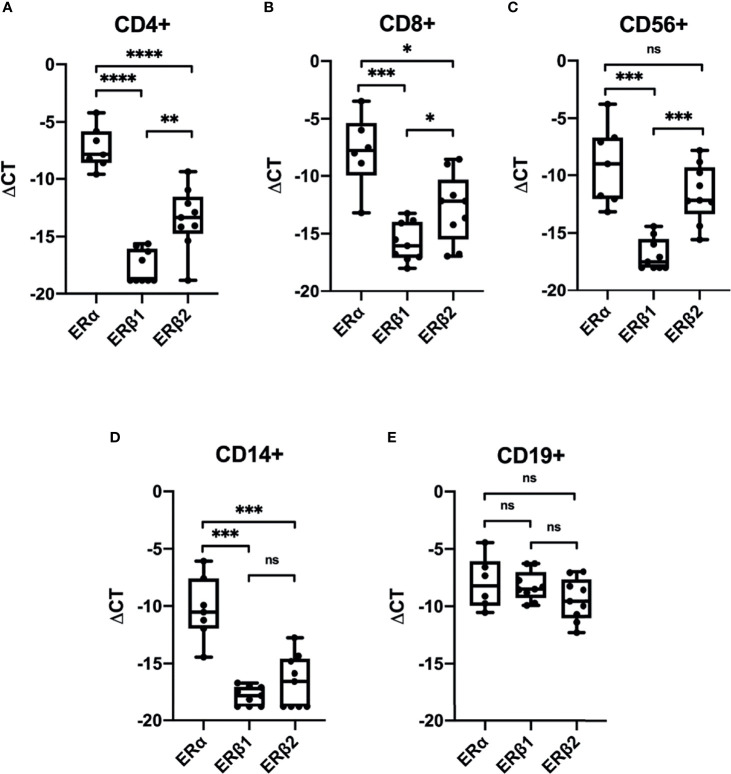
Expression of estrogen receptors in sorted PBMCs. Relative expression (ΔCT relative to GAPDH) of ERα (*ESR1*), ERβ1 (*ESR2_ERb1*) and ERβ2 (*ESR2_ERb2*) in CD4^+^ T-cells **(A)**, CD8^+^ T-cells **(B)**, CD56^+^ NK-cells **(C)**, CD14^+^ monocytes **(D)**, and CD19^+^ B-cells **(E)** from men, pre-MP and post-MP women (*n* = 6–9). *P* values were obtained using Welch’s t-test, *p < 0.05, **p < 0.01, ***p < 0.001, ****p < 0.0001, ns, not significant.

To investigate the presence of ERs in CD4^+^ T-cells, CD8^+^ T-cells, CD56^+^ NK-cells, CD14^+^ monocytes and CD19^+^ B-cells, we sorted PBMCs from healthy pre-MP, post-MP, and males by FACS (*n* = 9). ERα (*ESR1*) expression was found in all cell types (**Figures 1A–E**). The ERβ1 (*ESR2*_*ERb1*, RefSeq NM_001437) transcript was found in very small amounts in all cell types except in B-cells. Similarly, the ERβ splice variant ERβ2 (*ESR2*_*ERb2*, RefSeq NM_001291712) was also most abundant in B-cells but was also found in higher amounts in (CD4^+^ and CD8^+^) T-cells and NK-cells compared to ERβ1 (**Figures 1A–E**). In monocytes *ESR2_ERB1* and *ESR2_ERB2* was either very low or not detected (**Figure 1D**). The membrane-associated ER, GPER1, was only expressed in CD8^+^ T-cells, CD14^+^ monocytes, and CD19^+^ B-cells in relatively high amount (**Supplemental Figure 1A**). For comparison, the expression of the glucocorticoid receptor (GR) was found highly expressed in all cell types (**Supplemental Figure 1B**). Although underpowered, we could not detect any significant differences in ER distribution between cell types and between pre-MP and post-MP/men (**Supplemental Figure 2**). Our data demonstrate that the ERβ splice variant ERβ2 (*ESR2*_*ERb2*) is present in higher abundance than the full-length ERβ1 in most PBMC cell types.

### Effect of Menstrual Cycle on Sex Hormone Receptor and Inflammatory Gene Expression

**Figure 2:**
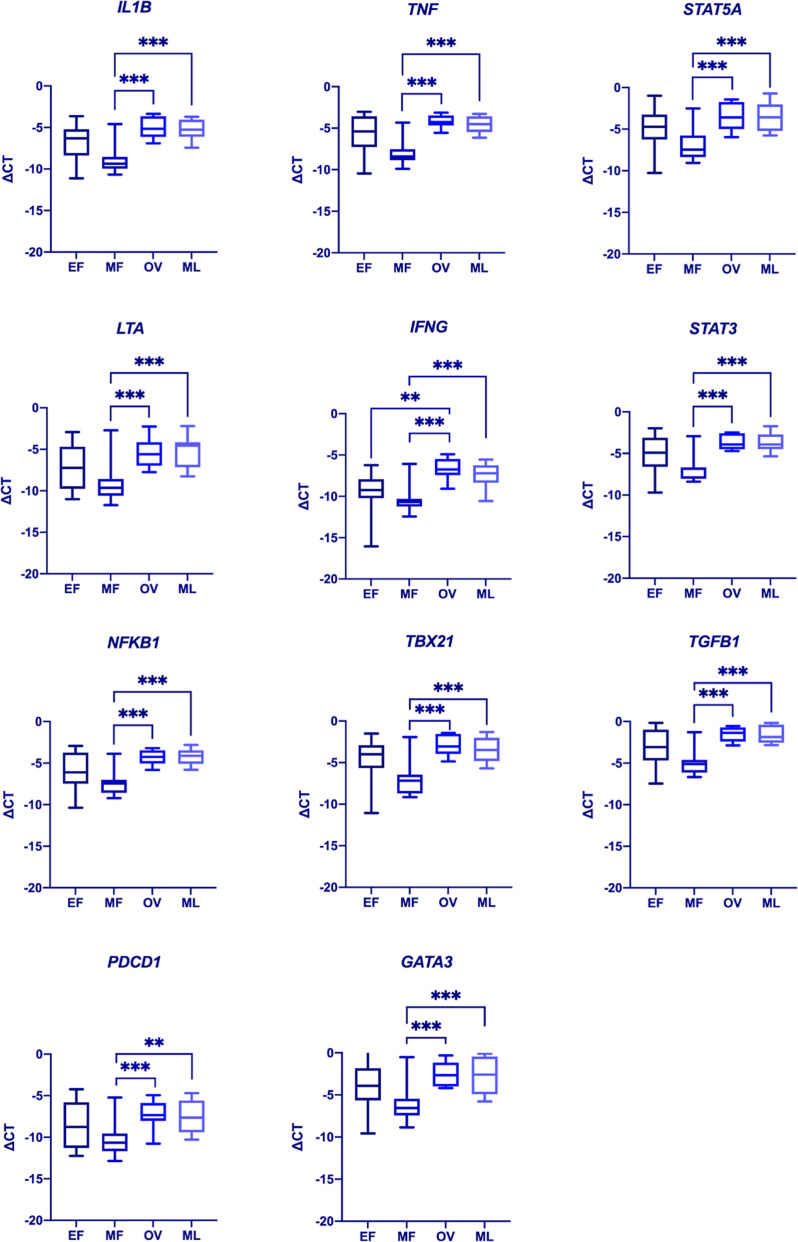
Differences in immune gene expression during the menstrual cycle. A generalized mixed model (GLMM, α = 0.002) was used to determine differences in gene expression between the various menstrual cycle phases (EF, early follicular phase; MF, mid follicular phase; Ov, ovulatory phase; ML, mid luteal phase). Significant differences could be observed for *IL1B*, *TNF*, *STAT5A*, *LTA*, *IFNG*, *STAT3*, *NFKB1*, *TBX21*, *TGFB1*, *PDCD1*, and *GATA3*. Data represent medians ±0.975 quartiles at df=9 in a t-distribution. Whiskers represent min and max values. **p = 0.001. ***p < 0.001. Complete list of *p*-values is included in **Supplemental Table 2**.

We next analyzed the expression of SHRs, and selected genes associated with immune response (**Supplemental Table 1**) in PBMCs during the menstrual cycle. To this end, we longitudinally sampled PBMCs from healthy Pre-MP women (*n* = 10) at 4 time-points representing early follicular (EF), mid-follicular (MF), ovulatory (OV) and mid luteal (ML) phases during the menstrual cycle and used a TLDA for gene expression analysis. We chose to use PBMCs rather than sorted cells to better illustrate the pooled expression profile of effector cells in the blood. A generalized linear mixed model (GLMM) based on ΔCT was performed to analyze the expression levels (**Supplemental Table 2**). Serum hormone levels were measured to confirm the hormone phases (**Supplemental Figure 3**). We could not detect any difference in SHR gene expression during the menstrual cycle for *AR, ESR1, ESR2_ERb1 or ESR2_ERb2* (*CYP19A1*, *IL17* and *PGR* were not expressed and omitted from the GLMM). However, several immune related genes (*GATA3, IFNG, IL1B, LTA, NFKB1, PDCD1, STAT3, STAT5A, TBX21, TGFB1, TNFA*) varied in their expression patterns during the menstrual cycle with significant differences comparing MF with ML phases and MF with OV phases (**Figure 2** and **Supplemental Table 2**). Interestingly, expression of both pro-inflammatory/T_H_1 response genes (*IL1B, TNF, LTA, IFNG, NFKB1, TBX21*, and *PDCD1*) and genes associated with T_H_2 response (*STAT3, STAT5A, TGFB1*, and *GATA3*) were significantly upregulated during OV and ML phases compared to the MF phase (**Figure 2**). No difference could be observed for GPER1 expression between the phases (**Supplemental Figure 4A**).

### Correlation Between SHR and Inflammatory Response Gene Expression

**Figure 3:**
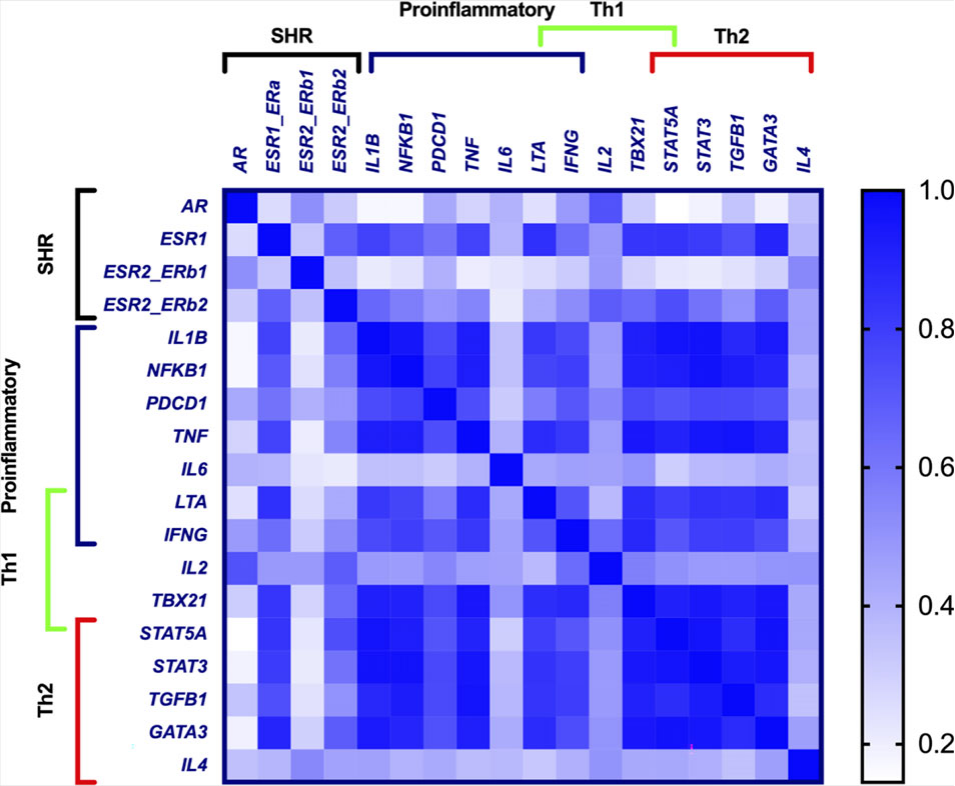
A heatmap of repeated measures correlation coefficients between gene expressions in PBMCs from pre-MP women. Increased blue color represent increased correlation (r-value → 1) (*n* = 10, each sampled 4 times). Proinflammatory genes, SHR genes, and genes associated with T_H_1, and T_H_2 response are indicated. *P*-values are listed in **Supplemental Table 3**.

To analyze if SHR expression was associated with specific sets of inflammatory response genes, we performed gene correlation analysis. *AR* and *ESR2_ERb1* correlated poorly with most genes studied (**Figure 3** and **Supplemental Table 3**). In contrast, *ESR1* and *ESR2_ERb2* had more similar correlation to each other and to most other genes studied. Most pronounced, *ESR1* had significant correlation with both proinflammatory T_H_1 and T_H_2-response genes (**Figure 3** and **Supplemental Table 3**).

### Correlation Between Hormone Levels and Immune Gene Expression in Pre-MP Women

**Figure 4:**
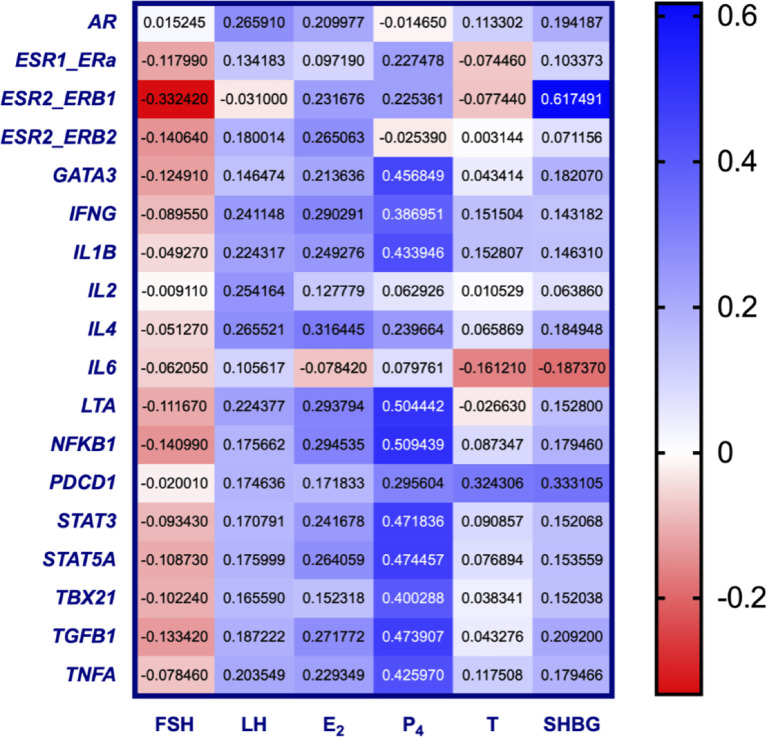
Heat map of repeated measures correlation coefficients between serum hormone levels and selected gene expression (-ΔCT) in PBMCs from pre-MP women. Increased blue color represent increased correlation (r-value → 1), increased red color represent increased anti-correlation (r-value → -1), and white represent no correlation (r = 0) (*n* = 10, each sampled 4 times). *P*-values are listed in **Supplemental Table 4** (α = 0.00032).

Correlation of serum levels of follicle stimulating hormone (FSH), luteinizing hormone (LH), estrogen (E_2_), progesterone (P_4_), and testosterone (T), as well as sex hormone binding globulin (SHBG), with the expression of inflammatory markers was analyzed in pre-MP women (*n* = 10) (**Figure 4** and **Supplemental Table 4**). Although we could not detect a significant correlation between any gene and hormone (at α = 0.00032), it is noteworthy that progesterone stood out with high r-numbers and/or low p-values to several genes (*NFKB1* [r = 0.509, *p* = 0.00342], *LTA* [r = 0.504, *p* = 0.00381], *STAT5A* [r = 0.474, *p* = 0.00700], *TGFB1* [r = 0.474, *p* = 0.00708], *STAT3* [r = 0.472, *p* = 0.00737], *GATA3* [r = 0.457, *p* = 0.00978], *IL1B* [r = 0.434, *p* = 0.0147], *TNFA* [r = 0.426, *p* = 0.0169], *TBX21* [r = 0.400, *p* = 0.0257], *IFNG* [r = 0.387 *p* = 0.0315]). This is potentially interesting for further investigations since the progesterone receptor (*PGR*) expression in PBMCs could not be detected, as mentioned above. Additionally, our data indicate that the levels of SHBG correlates positively with *ESR2_ERb1* (r = 0.617, *p* = 0.000215) (**Figure 4** and **Supplemental Table 4**). No significant correlation could be observed for GPER1 expression and hormone levels (**Supplemental Figure 4B**).

## Discussion

In this study we identify that ERα is the predominant estrogen receptor in PBMCs and that the expression of the ERβ alternative splice variant ERβ2 generally is more abundant than the full-length ERβ1 variant in PBMCs. Further, we demonstrate that the expression of several immune-related genes fluctuates in relation to the menstrual cycle. Using FACS to sort out CD4^+^ T-cells, CD8^+^ T-cells, CD56^+^ NK-cells, CD14^+^ monocytes, and CD19^+^ B-cells from PBMCs, we could identify that CD19^+^ B-cells have high expression of all ERs studied (ERα, ERβ1, ERβ2, and GPER1). In contrast, CD14^+^ monocytes have very low expression of ERβ1 and ERβ2, but high ERα and GPER1 expression. In addition, GPER1 was only found in CD8^+^ T-cells, CD14^+^ monocytes, and CD19^+^ B-cells with an overall high expression in these cell types. Taken together, the findings provide new information to better understand the interplay between sex hormones and immune responses.

We show that ERβ2 is significantly more abundant than ERβ1 (full length) in most immune cell subsets. Phiel *et al.* did previously report presence of both ERα and ERβ in PBMC (9), but they did not discriminate between ERβ isoforms. Importantly, ERβ2 does not bind E_2_ but can dimerize with both ERα and ERβ1, to inhibit their transcriptional activity. Oppositely to the present study, it was earlier described that patients with chronic lymphocytic leukemia (CLL) had higher levels of ERβ2 in PBMCs compared to healthy donors where ERβ1 dominated (16). However, that study was performed by assessing ERβ2-protein staining (using immunocytochemistry, ICC), rather than quantifying absolute expression. In addition, the median age among CLL patients (68 years) and healthy donors (43 years) differed, so an age difference in ERβ1/ERβ2 distribution cannot be excluded. Although we did not analyze ERα splice variants, the study by Stygar and colleagues (17) detected some expression of ERα splice variants in PBMCs and that this expression could vary with the menstrual cycle. However, the samples used in that study were derived from 6 pre-MP women in the follicular phase, and 3 in the secretory phase (i.e., the individuals were not sampled repeatedly) so an inter-individual difference cannot be excluded. Clearly, more studies are needed to determine ERβ2’s role in relation to other ER variants, sex hormone levels, and age.

Furthermore, we show that the expression of several immune genes in bulk PBMCs (*GATA3, IFNG, IL1B, LTA, NFKB1, PDCD1, STAT3, STAT5A, TBX21, TGFB1, TNFA*) differed between phases of the menstrual cycle. We did not observe differences in SHR expression patterns between the phases, possibly this is linked to an important limitation of our study which is the low number of participants. In addition, the low amount of sample material prevented sorting out the cell populations for TLDA analysis by FACS. Nevertheless, differences in immune gene expression patterns were significant and we speculate that even more immune related genes could potentially be found by increasing the participant number. *IFNG, TNFA* and *IL1B* are all genes that encode proinflammatory responses. NFκB is an inducible transcription factor that can be regulated by steroid hormone signaling (18), and controls expression of several stress response genes and genes associated with development of innate immunity. Among NFκB target genes are regulators of inflammatory cytokines, cell survival, proliferation and cell surface proteins (18–20). NFκB activity has also been suggested to play a significant role for female fertility by participating in angiogenesis during corpus luteum formation, endometrial implantation and indeed also for the T_H_1-T_H_2 immune response shift seen during the menstrual cycle (important for the tolerance of the semi-allogenic blastocyst implantation) (21).

Additionally, we demonstrate that the expression of *GATA3* and *TBX21* are both fluctuating during the menstrual cycle. *TBX21* (encoding for T-bet) and *GATA3* are both key transcription factors for T_H_1 and T_H_2 immune response respectively. It should be noted that the distinction of T_H_-cells into T_H_1- and T_H_2-cells, although still widely in use, have been questioned since the discovery of further T_H_-subsets (as T_H_17 and Treg-cells) (22–24). Prior studies suggest that the immune response shifts from a T_H_1 to T_H_2 response over the menstrual cycle (7). Although the expression of *GATA3* and *TBX21* differs over the menstrual cycle, our data do not support a T_H_1-T_H_2 shift, as both *GATA3* and *TBX21* are highly expressed during the latter part of the cycle (OV and ML phases). LTA (TNF-β) is also related to T_H_1 response, as it is secreted from T_H_1 but not T_H_2 cells. A different experimental design including more participants (and more frequent sampling during the menstrual cycle) may reveal a more fine-tuned regulation of *GATA3* and *TBX21*. The same expression pattern is seen with *PDCD1* and *TGFB1* which are significantly more expressed during OV and ML phases. *PDCD1* and *TGFB1* are both related to immune tolerance. PD-1 may have implications for development of autoimmunity, chronic infectious diseases and several types of cancer, and expression of its gene *PDCD1* is related to sex hormones, particularly E_2_ (25). TGF-β, stimulates differentiation of CD4^+^ T-cells to Treg-cells and has an inhibitory effect on B-cell proliferation. Previous studies have showed a positive correlation between E_2_ and Treg numbers during the menstrual cycle (26).

Like the genes mentioned above, *STAT3* and *STAT5A* are also significantly higher expressed during OV and ML phases. STAT5 has previously been associated with sex differences in liver metabolism (27) and pulmonary hypertension (28), both with a proposed neuroendocrine regulation through hypothalamus-growth hormone-STAT5 axis. In addition, STAT5 has an important role in the priming of CD4^+^ T-cells for T_H_1, T_H_2 and T_H_9 development (29). STAT3 has on the other hand been pointed out as factor of major importance in the pathogenesis of gastrointestinal bacterial infections and cancer development along with viral infectious diseases (HBV, HCV and HPV) which in turn may drive cancer development (30).

We further found that ERβ1 (*ESR_ERB1*) stood out as significantly positively correlated to SHBG levels. SHBG is a circulating glycoprotein synthesized and secreted by the liver, with a main function of transporting sex steroids, mainly testosterone, in the circulation, thereby modulating sex hormone bioavailability. In a study by Maggio *et al.* (31) on postmenopausal women, SHBG was negatively correlated to inflammatory markers such as C-reactive protein (CRP), IL-6 and soluble IL-6 receptor (sIL-6r). In the same study E_2_ was positively correlated to CRP and IL-6 (but not sIL-6r). It is possible that the opposite correlation between E_2_ and SHBG on inflammation might be due to increased expression of ERβ1 which oppose the action of ERα.

In this study, we could neither observe a general immunostimulatory nor an immunosuppressive signature that could be linked to the different phases of the menstrual cycle. Rather, both immunostimulatory and immunosuppressive response genes were upregulated during ovulation and the mid luteal phase. Generally, E_2_ is immunostimulatory while progesterone (P_4_) and testosterone have immunosuppressive properties (described in detail in e.g (2).,). Testosterone will e.g., decrease humoral immunity (increase B-cell apoptosis of immature B-cells). P_4_ will decrease hypermutation and class-switch of B-cells and E_2_ will decrease B-cell apoptosis, promote class-switching and hypermutation and increase the number of autoreactive antibodies (32).

An increase in P_4_ in the luteal phase is attributed a general suppressive effect on the innate immune response by e.g., decreasing the production of proinflammatory cytokines (33). In the present study, gene-hormone correlations were not clear-cut, the P_4_-levels might be involved in the regulation of several immune response genes, but our statistical evaluations did not provide a significant signal (**Figure 4** and **Supplemental Table 4**). PGR is reportedly present in immune cells (34). Recent findings by Hierweger and coworkers (35), however, question its presence in T-cells suggesting that P_4_ may signal through the glucocorticoid receptor (GR) in these cells. The suggestion by Hierweger is in line with our data since PGR was not expressed in our material. Although GR was not part of our qPCR array, we could detect high GR levels in all sorted PBMCs (**Supplemental Figure 1**). Therefore, we hypothesize that any correlation of P_4_ with gene expression in PBMC is indirect, e.g., through GR. Future studies including GR could help answering these questions.

In conclusion, we demonstrate that several key immune related genes in PBMCs fluctuate in their expression according to the phase of the menstrual cycle. This includes both proinflammatory, T_H_1- and T_H_2-response genes. In addition, this paper illustrates that mRNA for ERβ2 is more abundant than ERβ1 in PBMCs, which suggests that ERβ2 may play a more prominent role than previously thought in the immune response. Our study provides evidence that the menstrual cycle can influence the immune response. Larger studies enrolling pre-MP women sampled over more timepoints of the menstrual cycle and including more ER splice variants and inflammatory genes in sorted PBMCs are warranted. In the end, such studies may provide information that allows for the development of personalized immune treatments to the benefit of both pre-MP women, post-MP women and men.

## Data Availability Statement

The original contributions presented in the study are included in the article/**Supplementary Material**. Further inquiries can be directed to the corresponding author.

## Ethics Statement

The studies involving human participants were reviewed and approved by Swedish Research Council (Dnr: Ö 24-2009). The patients/participants provided their written informed consent to participate in this study.

## Author Contributions

PB: Conceived the study, performed the experiments, analyzed data, and wrote the manuscript. B-ML: Conceived the study, collected the patient material, analyzed data, and wrote the manuscript. PF: Analyzed the data and wrote the manuscript. MS: Performed the experiments. J-AG: Financed the experiments and contributed with laboratory equipment, and wrote the manuscript. AJ: Conceived the study, analyzed data, and wrote the manuscript. IN: Conceived the study, performed the experiments, analyzed data, and wrote the manuscript.

## Funding

This study was supported by the Karolinska Institutet (FS-2018:0007), Region Västerbotten (RV-866221) and Folksams Forskningsstiftelse. J-AG is grateful to the Robert A. Welch Foundation for a grant (E-0004).

## Conflict of Interest

The authors declare that the research was conducted in the absence of any commercial or financial relationships that could be construed as a potential conflict of interest.

The handling editor declared a shared affiliation with several of the authors, PB, B-ML, MS, J-AG, and IN, at time of review.

## Publisher’s Note

All claims expressed in this article are solely those of the authors and do not necessarily represent those of their affiliated organizations, or those of the publisher, the editors and the reviewers. Any product that may be evaluated in this article, or claim that may be made by its manufacturer, is not guaranteed or endorsed by the publisher.
