# Supplementary material for: Expression of Sex Hormone Receptor and Immune Response Genes in Peripheral Blood Mononuclear Cells During the Menstrual Cycle
Source: Front Endocrinol (Lausanne). 2021 Sep 22;12:721813. doi: 10.3389/fendo.2021.721813 (PMC8493253; doi:10.3389/fendo.2021.721813)
Supplement: Supplementary file 1 [file DataSheet_1.pdf]

Supplemental Figure 1.

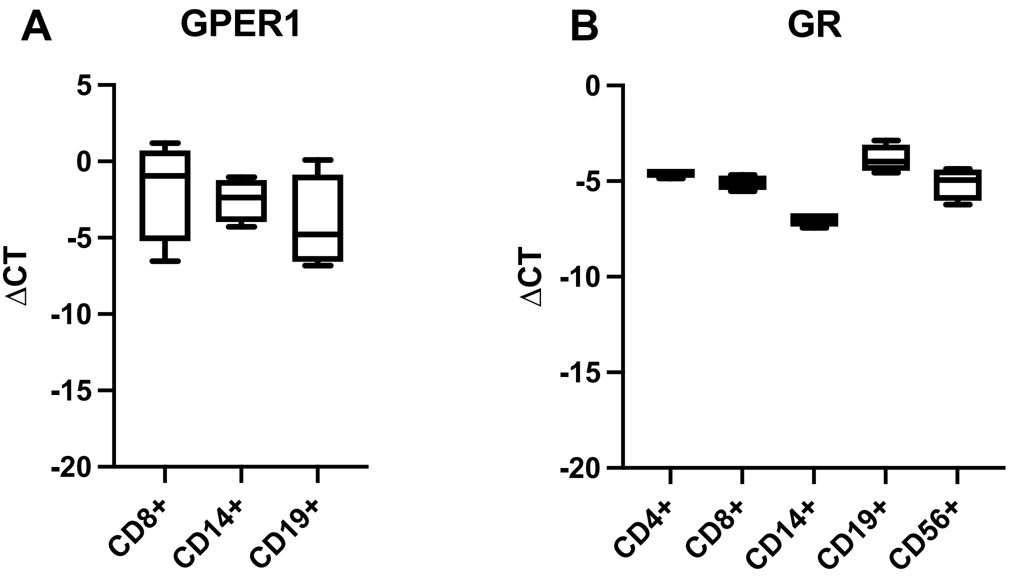

Supplemental Figure 1. Expression of the (**A**) GPER1 and (**B**) glucocorticoid receptor (GR) in sorted PBMCs. Relative expression (ΔCT relative to GAPDH) in $CD4^+$ T-cells, $CD8^+$ T-cells, $CD56^+$ NK-cells, $CD14^+$ monocytes, and $CD19^+$ B-cells from men, pre-MP and post-MP women (n = 4). GPER1 was only detected in $CD8^+$ T-cells, CD14+ monocytes, and $CD19^+$ B cells.
