# Supplementary material for: Expression of Sex Hormone Receptor and Immune Response Genes in Peripheral Blood Mononuclear Cells During the Menstrual Cycle
Source: Front Endocrinol (Lausanne). 2021 Sep 22;12:721813. doi: 10.3389/fendo.2021.721813 (PMC8493253; doi:10.3389/fendo.2021.721813)
Supplement: Supplementary file 2 [file DataSheet_2.pdf]

Supplemental Figure 2.

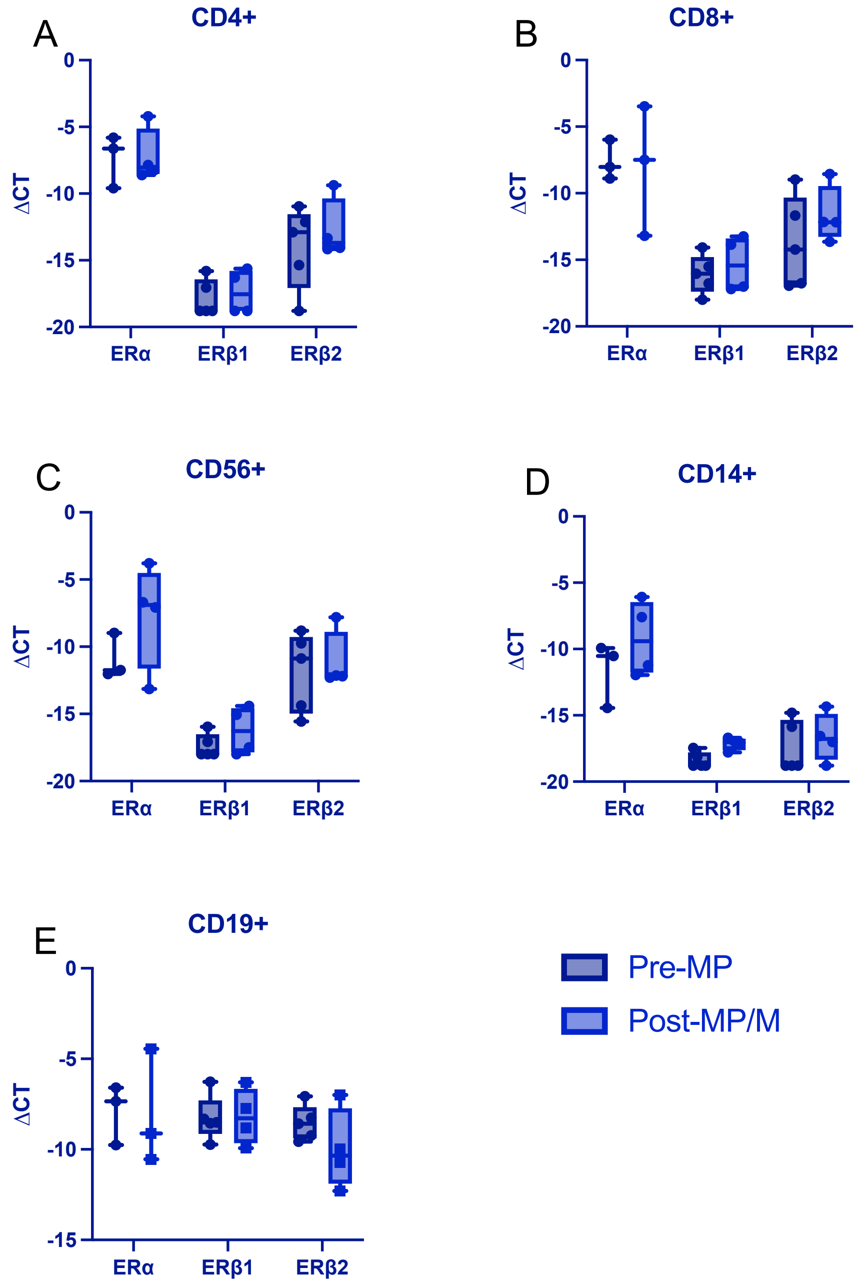

Supplemental Figure 2. Comparison of estrogen receptor expression in sorted PBMCs between pre-menopausal women (pre-MP, *n* = 3-5) and post-menopausal women and men (post-MP/M, *n* = 3-4). Relative expression (ΔCT relative to GAPDH) of ERα (*ESR1*), ERβ1 (*ESR2_ERb1*) and ERβ2 (*ESR2_ERb2*) in CD4^{+} T-cells (**A**), CD8^{+} T-cells (B), CD56^{+} NK-cells (**C**), CD14^{+} monocytes (**D**), and CD19^{+} B-cells (**E**). No statistical significance was detected between Pre-MP and post-MP/M (2-way ANOVA).
