# Supplementary material for: Expression of Sex Hormone Receptor and Immune Response Genes in Peripheral Blood Mononuclear Cells During the Menstrual Cycle
Source: Front Endocrinol (Lausanne). 2021 Sep 22;12:721813. doi: 10.3389/fendo.2021.721813 (PMC8493253; doi:10.3389/fendo.2021.721813)
Supplement: Supplementary file 3 [file DataSheet_3.pdf]

Supplemental Figure 3.

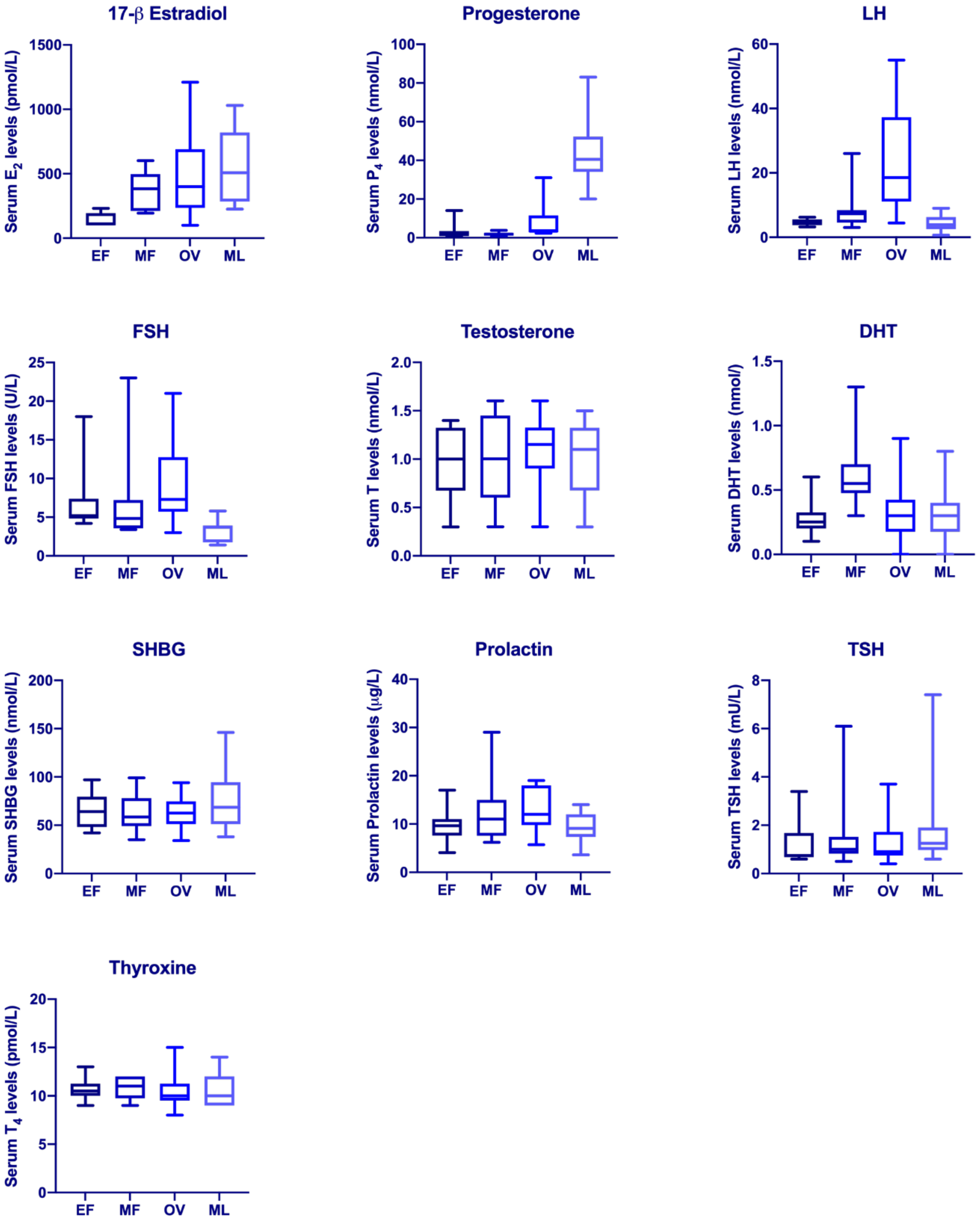

Supplemental Figure 3. Serum hormone levels across menstrual cycle phases (EF, early follicular; MF, mid-follicular, OV, ovulatory; ML, mid luteal) from pre-MP women (*n* = 10). Data represent medians with upper and lower confidence intervals, and min. and max. values. Hormones sampled: estradiol (E2), progesterone, (P4), luteinizing hormone (LH), follicle stimulating hormone (FSH), testosterone, dihydrotestosterone (DHT), prolactin, thyroid-stimulating hormone (TSH), and thyroxine (T4). In addition, the levels of sex hormone-binding globulin (SHBG) were sampled.
