# Supplementary material for: Expression of Sex Hormone Receptor and Immune Response Genes in Peripheral Blood Mononuclear Cells During the Menstrual Cycle
Source: Front Endocrinol (Lausanne). 2021 Sep 22;12:721813. doi: 10.3389/fendo.2021.721813 (PMC8493253; doi:10.3389/fendo.2021.721813)
Supplement: Supplementary file 4 [file DataSheet_4.pdf]

Supplemental figure 4.

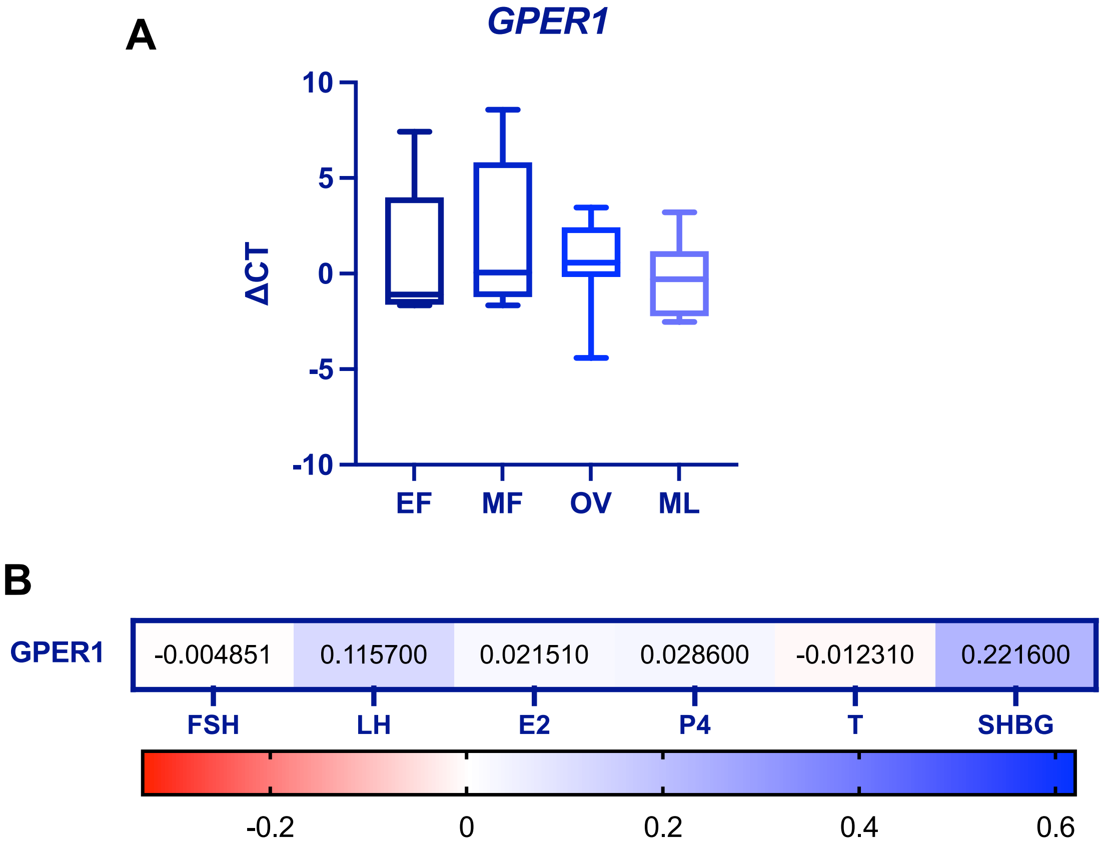

Supplemental figure 4. Differences in *GPER1* expression in PBMCs during the menstrual cycle and correlation to serum hormone levels. (**A**) One-way ANOVA analysis was used to determine differences in *GPER1* gene expression (as measured by qPCR) between the various menstrual cycle phases (EF, early follicular phase; MF, mid follicular phase; OV, ovulatory phase; ML, mid luteal phase). No significant differences could be observed. Data represent medians ±0.975 quartiles, $n = 10$ women. Whiskers represent min and max values. (**B**) Heat map of repeated measures correlation coefficients between serum hormone levels and *GPER1* gene expression (-ΔCT) in PBMCs from pre-MP women. Increased blue color represent increased correlation (r-value → 1), increased red color represent increased anti-correlation (r-value → -1), and white represent no correlation (r = 0). No significant correlation was found ($n = 10$, women sampled over the 4 menstrual cycle phases).
