# Supplementary material for: Expression of Sex Hormone Receptor and Immune Response Genes in Peripheral Blood Mononuclear Cells During the Menstrual Cycle
Source: Front Endocrinol (Lausanne). 2021 Sep 22;12:721813. doi: 10.3389/fendo.2021.721813 (PMC8493253; doi:10.3389/fendo.2021.721813)
Supplement: Supplementary file 5 [file DataSheet_5.pdf]

Supplemental table 1. Genes included in Custom Taqman Array (Thermo Fisher).

Gene name	NCBI Gene ID	Probe Assay ID
IFNG	3458	Hs00989291_m1
IL2	3558	Hs00174114_m1
LTA	4049	Hs04188773_g1
IL5	3567	Hs01548712_g1
TBX21	30009	Hs00894392_m1
IL4	3565	Hs00174122_m1
IL13	3596	Hs00174379_m1
GATA3	2625	Hs00231122_m1
IL1B	3553	Hs01555410_m1
IL12B	3593	Hs01011518_m1
TNF	7124	Hs00174128_m1
IL6	3569	Hs00174131_m1
IL3	3562	Hs00174117_m1
IL10	3586	Hs00961622_m1
TGFB1	7040	Hs99999918_m1
IL17A	3605	Hs00174383_m1
PGR	5241	Hs01556702_m1
AR	367	Hs00171172_m1
CYP19A1	1588	Hs00903411_m1
ESR1	2099	Hs00174860_m1
ESR2_ERb1	2100	Hs01100359_m1
ESR2_ERb2	2100	Hs01105520_m1
GREB1	9687	Hs00536409_m1
NFKB1	4790	Hs00765730_m1
CXCL8	3576	Hs00174103_m1
STAT5A	6776	Hs00559637_g1
STST3	6774	Hs00374280_m1
CD274	29126	Hs00204257_m1
PDCD1	5133	Hs01550088_m1
GAPDH	2597	Hs99999905_m1
