# Supplementary material for: Expression of Sex Hormone Receptor and Immune Response Genes in Peripheral Blood Mononuclear Cells During the Menstrual Cycle
Source: Front Endocrinol (Lausanne). 2021 Sep 22;12:721813. doi: 10.3389/fendo.2021.721813 (PMC8493253; doi:10.3389/fendo.2021.721813)
Supplement: Supplementary file 6 [file DataSheet_6.pdf]

Supplemental table 2. Gene expression in PBMCs from pre-MP women (n=10, sampled 4 times, df = 27) related to phase of the menstrual cycle. EF = Early Follicular, MF = Mid follicular, OV = Ovulatory, ML = Mid Luteal phase. df = 27. LMM analysis was used to determine *p*-values.

Phases	Gene	estimate	SE	t-ratio	*p*-value	Phases	Gene	estimate	SE	t-ratio	*p*-value
EF vs MF	*AR*	-0.989	2.256	-0.439	0.971	EF vs MF	*IL6*	-2.336	1.98	-1.18	0.644
EF vs OV	*AR*	3.431	2.256	1.521	0.44	EF vs OV	*IL6*	2.6	1.98	1.313	0.563
EF vs ML	*AR*	2.19	2.256	0.971	0.767	EF vs ML	*IL6*	-0.546	1.98	-0.276	0.992
MF vs OV	*AR*	4.421	2.256	1.959	0.228	MF vs OV	*IL6*	4.936	1.98	2.493	0.084
MF vs ML	*AR*	3.179	2.256	1.409	0.505	MF vs ML	*IL6*	1.79	1.98	0.904	0.803
OV vs ML	*AR*	-1.241	2.256	-0.55	0.946	OV vs ML	*IL6*	-3.146	1.98	-1.589	0.402
EF vs MF	*ESR1*	-0.989	2.256	-0.439	0.971	EF vs MF	*LTA*	-1.902	0.679	-2.799	0.044
EF vs OV	*ESR1*	3.431	2.256	1.521	0.44	EF vs OV	*LTA*	1.806	0.679	2.658	0.059
EF vs ML	*ESR1*	2.19	2.256	0.971	0.767	EF vs ML	*LTA*	1.96	0.679	2.885	0.036
MF vs OV	*ESR1*	4.421	2.256	1.959	0.228	**MF vs OV**	***LTA***	**3.708**	**0.679**	**5.456**	**<0.001**
MF vs ML	*ESR1*	3.179	2.256	1.409	0.505	**MF vs ML**	***LTA***	**3.862**	**0.679**	**5.684**	**<0.001**
OV vs ML	*ESR1*	-1.241	2.256	-0.55	0.946	OV vs ML	*LTA*	0.155	0.679	0.227	0.996
EF vs MF	*ESR2_ERb1*	-0.499	1.55	-0.322	0.988	EF vs MF	*NFKB1*	-1.501	0.518	-2.898	0.035
EF vs OV	*ESR2_ERb1*	0.109	1.55	0.07	1	EF vs OV	*NFKB1*	1.697	0.518	3.275	0.014
EF vs ML	*ESR2_ERb1*	2.378	1.55	1.534	0.432	EF vs ML	*NFKB1*	1.73	0.518	3.339	0.012
MF vs OV	*ESR2_ERb1*	0.608	1.55	0.392	0.979	**MF vs OV**	***NFKB1***	**3.197**	**0.518**	**6.173**	**<0.001**
MF vs ML	*ESR2_ERb1*	2.877	1.55	1.856	0.27	**MF vs ML**	***NFKB1***	**3.23**	**0.518**	**6.237**	**<0.001**
OV vs ML	*ESR2_ERb1*	2.269	1.55	1.464	0.473	OV vs ML	*NFKB1*	0.033	0.518	0.064	1
EF vs MF	*ESR2_ERb2*	-2.227	1.102	-2.021	0.206	EF vs MF	*PDCD1*	-1.693	0.648	-2.612	0.065
EF vs OV	*ESR2_ERb2*	2.134	1.102	1.936	0.237	EF vs OV	*PDCD1*	1.368	0.648	2.111	0.175
EF vs ML	*ESR2_ERb2*	0.778	1.102	0.706	0.894	EF vs ML	*PDCD1*	1.086	0.648	1.675	0.356
MF vs OV	*ESR2_ERb2*	4.361	1.102	3.956	0.003	**MF vs OV**	***PDCD1***	**3.061**	**0.648**	**4.723**	**<0.001**
MF vs ML	*ESR2_ERb2*	3.005	1.102	2.727	0.051	**MF vs ML**	***PDCD1***	**2.779**	**0.648**	**4.287**	**0.001**
OV vs ML	*ESR2_ERb2*	-1.355	1.102	-1.23	0.614	OV vs ML	*PDCD1*	-0.283	0.648	-0.436	0.972

EF vs MF	*GATA3*	-2.158	0.655	-3.295	0.014	EF vs MF	*STAT3*	-1.796	0.563	-3.193	0.018
EF vs OV	*GATA3*	1.471	0.655	2.245	0.137	EF vs OV	*STAT3*	1.39	0.563	2.47	0.088
EF vs ML	*GATA3*	1.409	0.655	2.151	0.163	EF vs ML	*STAT3*	1.379	0.563	2.451	0.091
**MF vs OV**	***GATA3***	**3.629**	**0.655**	**5.541**	**<0.001**	**MF vs OV**	***STAT3***	**3.186**	**0.563**	**5.663**	**<0.001**
**MF vs ML**	***GATA3***	**3.567**	**0.655**	**5.446**	**<0.001**	**MF vs ML**	***STAT3***	**3.175**	**0.563**	**5.644**	**<0.001**
OV vs ML	*GATA3*	-0.062	0.655	-0.095	1	OV vs ML	*STAT3*	-0.011	0.563	-0.019	1
EF vs MF	*IFNG*	-0.918	0.644	-1.425	0.495	EF vs MF	*STAT5A*	-1.923	0.572	-3.364	0.012
**EF vs OV**	***IFNG***	**2.87**	**0.644**	**4.457**	**0.001**	EF vs OV	*STAT5A*	1.407	0.572	2.46	0.09
EF vs ML	*IFNG*	2.122	0.644	3.295	0.014	EF vs ML	*STAT5A*	1.413	0.572	2.472	0.087
**MF vs OV**	***IFNG***	**3.787**	**0.644**	**5.882**	**<0.001**	**MF vs OV**	***STAT5A***	**3.33**	**0.572**	**5.824**	**<0.001**
**MF vs ML**	***IFNG***	**3.039**	**0.644**	**4.72**	**<0.001**	**MF vs ML**	***STAT5A***	**3.337**	**0.572**	**5.836**	**<0.001**
OV vs ML	*IFNG*	-0.748	0.644	-1.162	0.655	OV vs ML	*STAT5A*	0.007	0.572	0.012	1
EF vs MF	*IL1B*	-2.095	0.561	-3.734	0.005	EF vs MF	*TBX21*	-2.374	0.656	-3.617	0.006
EF vs OV	*IL1B*	1.863	0.561	3.32	0.013	EF vs OV	*TBX21*	1.63	0.656	2.484	0.085
EF vs ML	*IL1B*	1.622	0.561	2.89	0.036	EF vs ML	*TBX21*	1.17	0.656	1.782	0.303
**MF vs OV**	***IL1B***	**3.959**	**0.561**	**7.054**	**<0.001**	**MF vs OV**	***TBX21***	**4.004**	**0.656**	**6.101**	**<0.001**
**MF vs ML**	***IL1B***	**3.717**	**0.561**	**6.623**	**<0.001**	**MF vs ML**	***TBX21***	**3.543**	**0.656**	**5.399**	**<0.001**
OV vs ML	*IL1B*	-0.242	0.561	-0.431	0.973	OV vs ML	*TBX21*	-0.461	0.656	-0.702	0.896
EF vs MF	*IL2*	-0.297	1.822	-0.163	0.998	EF vs MF	*TGFB1*	-1.949	0.552	-3.53	0.008
EF vs OV	*IL2*	4.372	1.822	2.4	0.101	EF vs OV	*TGFB1*	1.574	0.552	2.851	0.039
EF vs ML	*IL2*	2.485	1.822	1.364	0.532	EF vs ML	*TGFB1*	1.563	0.552	2.832	0.04
MF vs OV	*IL2*	4.669	1.822	2.563	0.072	**MF vs OV**	***TGFB1***	**3.523**	**0.552**	**6.381**	**<0.001**
MF vs ML	*IL2*	2.783	1.822	1.527	0.436	**MF vs ML**	***TGFB1***	**3.512**	**0.552**	**6.362**	**<0.001**
OV vs ML	*IL2*	-1.887	1.822	-1.036	0.73	OV vs ML	*TGFB1*	-0.01	0.552	-0.019	1
EF vs MF	*IL4*	-0.765	1.79	-0.427	0.973	EF vs MF	*TNFA*	-2.183	0.581	-3.76	0.004
EF vs OV	*IL4*	2.643	1.79	1.476	0.465	EF vs OV	*TNFA*	1.655	0.581	2.851	0.039
EF vs ML	*IL4*	2.938	1.79	1.641	0.373	EF vs ML	*TNFA*	1.327	0.581	2.286	0.126
MF vs OV	*IL4*	3.407	1.79	1.903	0.25	**MF vs OV**	***TNFA***	**3.838**	**0.581**	**6.61**	**<0.001**
MF vs ML	*IL4*	3.703	1.79	2.069	0.189	**MF vs ML**	***TNFA***	**3.51**	**0.581**	**6.046**	**<0.001**
OV vs ML	*IL4*	0.296	1.79	0.165	0.998	OV vs ML	*TNFA*	-0.328	0.581	-0.564	0.942
