# Supplementary material for: Expression of Sex Hormone Receptor and Immune Response Genes in Peripheral Blood Mononuclear Cells During the Menstrual Cycle
Source: Front Endocrinol (Lausanne). 2021 Sep 22;12:721813. doi: 10.3389/fendo.2021.721813 (PMC8493253; doi:10.3389/fendo.2021.721813)
Supplement: Supplementary file 7 [file DataSheet_7.pdf]

Supplemental Table 3. Repeated measures correlation coefficients (α = 0.0002) in PBMCs from pre-MP women (*n* = 10, each sampled 4 times). Lower and upper confidence intervals (CI), r- and *p*-values are shown for indicated gene 1 vs. gene 2.

Gene 1	Gene 2	r	Lower CI	Upper CI	*p*-value
AR	*ESR1_ERa*	0.367	1E-03	0.647	0.042
AR	*ESR2_ERb1*	0.632	0.346	0.811	1E-04
AR	*ESR2_ERb2*	0.571	0.258	0.775	8E-04
AR	*GATA3*	0.231	-0.148	0.551	0.212
AR	*IFNG*	0.499	0.162	0.732	0.004
AR	*IL1B*	0.237	-0.141	0.556	0.199
AR	*IL2*	0.828	0.662	0.916	9E-09
AR	*IL4*	0.566	0.251	0.772	9E-04
AR	*IL6*	0.364	-0.003	0.644	0.044
AR	*LTA*	0.292	-0.083	0.595	0.111
AR	*NFKB1*	0.209	-0.171	0.535	0.259
AR	*PDCD1*	0.457	0.108	0.705	0.01
AR	*STAT3*	0.187	-0.192	0.518	0.313
AR	*STAT5A*	0.172	-0.208	0.506	0.356
AR	*TBX21*	0.302	-0.073	0.602	0.099
AR	*TGFB1*	0.3	-0.075	0.6	0.101
AR	*TNFA*	0.26	-0.118	0.572	0.158
ESR1_ERa	*ESR2_ERb1*	0.222	-0.158	0.544	0.231
ESR1_ERa	*ESR2_ERb2*	0.71	0.464	0.854	8E-06
ESR1_ERa	*GATA3*	0.869	0.738	0.937	2E-10
ESR1_ERa	*IFNG*	0.721	0.482	0.86	5E-06
ESR1_ERa	*IL1B*	0.791	0.598	0.898	1E-07
ESR1_ERa	*IL2*	0.573	0.261	0.776	8E-04
ESR1_ERa	*IL4*	0.424	0.068	0.684	0.017
ESR1_ERa	*IL6*	0.628	0.339	0.808	2E-04
ESR1_ERa	*LTA*	0.817	0.642	0.911	2E-08
ESR1_ERa	*NFKB1*	0.808	0.627	0.906	4E-08
ESR1_ERa	*PDCD1*	0.669	0.401	0.832	4E-05
ESR1_ERa	*STAT3*	0.835	0.675	0.92	5E-09
ESR1_ERa	*STAT5A*	0.826	0.66	0.916	1E-08
ESR1_ERa	*TBX21*	0.843	0.689	0.924	3E-09
ESR1_ERa	*TGFB1*	0.837	0.678	0.921	4E-09
ESR1_ERa	*TNFA*	0.836	0.677	0.921	5E-09
ESR2_ERb1	*ESR2_ERb2*	0.268	-0.11	0.578	0.145
ESR2_ERb1	*GATA3*	0.136	-0.242	0.479	0.465
ESR2_ERb1	*IFNG*	0.234	-0.145	0.553	0.205
ESR2_ERb1	*IL1B*	0.036	-0.335	0.397	0.846
ESR2_ERb1	*IL2*	0.496	0.159	0.73	0.005
ESR2_ERb1	*IL4*	0.54	0.216	0.757	0.002
ESR2_ERb1	*IL6*	0.252	-0.126	0.566	0.171
ESR2_ERb1	*LTA*	0.206	-0.174	0.532	0.266
ESR2_ERb1	*NFKB1*	0.064	-0.309	0.421	0.731
ESR2_ERb1	*PDCD1*	0.261	-0.117	0.572	0.157
ESR2_ERb1	*STAT3*	0.026	-0.344	0.389	0.89
ESR2_ERb1	*STAT5A*	0.023	-0.347	0.386	0.904
ESR2_ERb1	*TBX21*	0.178	-0.202	0.511	0.339
ESR2_ERb1	*TGFB1*	0.14	-0.239	0.482	0.452
ESR2_ERb1	*TNFA*	0.096	-0.281	0.447	0.609

ESR2_ERb2	*GATA3*	0.603	0.304	0.794	3E-04
ESR2_ERb2	*IFNG*	0.658	0.384	0.825	6E-05
ESR2_ERb2	*IL1B*	0.605	0.306	0.795	3E-04
ESR2_ERb2	*IL2*	0.627	0.338	0.808	2E-04
ESR2_ERb2	*IL4*	0.306	-0.068	0.605	0.094
ESR2_ERb2	*IL6*	0.425	0.07	0.685	0.017
ESR2_ERb2	*LTA*	0.573	0.261	0.776	8E-04
ESR2_ERb2	*NFKB1*	0.602	0.302	0.794	3E-04
ESR2_ERb2	*PDCD1*	0.563	0.248	0.771	1E-03
ESR2_ERb2	*STAT3*	0.576	0.265	0.778	7E-04
ESR2_ERb2	*STAT5A*	0.591	0.287	0.787	5E-04
ESR2_ERb2	*TBX21*	0.623	0.332	0.806	2E-04
ESR2_ERb2	*TGFB1*	0.65	0.372	0.821	8E-05
ESR2_ERb2	*TNFA*	0.628	0.339	0.808	2E-04
GATA3	*IFNG*	0.86	0.72	0.932	6E-10
GATA3	*IL1B*	0.934	0.862	0.969	2E-14
GATA3	*IL2*	0.422	0.066	0.683	0.018
GATA3	*IL4*	0.436	0.083	0.692	0.014
GATA3	*IL6*	0.592	0.288	0.788	5E-04
GATA3	*LTA*	0.933	0.86	0.968	2E-14
GATA3	*NFKB1*	0.968	0.933	0.985	5E-19
GATA3	*PDCD1*	0.772	0.566	0.887	4E-07
GATA3	*STAT3*	0.976	0.949	0.989	1E-20
GATA3	*STAT5A*	0.967	0.93	0.984	1E-18
GATA3	*TBX21*	0.972	0.941	0.987	8E-20
GATA3	*TGFB1*	0.966	0.927	0.984	2E-18
GATA3	*TNFA*	0.966	0.929	0.984	1E-18
IFNG	*IL1B*	0.836	0.676	0.92	5E-09
IFNG	*IL2*	0.644	0.363	0.817	9E-05
IFNG	*IL4*	0.569	0.255	0.774	8E-04
IFNG	*IL6*	0.629	0.341	0.809	2E-04
IFNG	*LTA*	0.855	0.712	0.93	9E-10
IFNG	*NFKB1*	0.872	0.744	0.939	2E-10
IFNG	*PDCD1*	0.765	0.554	0.884	5E-07
IFNG	*STAT3*	0.835	0.675	0.92	5E-09
IFNG	*STAT5A*	0.823	0.654	0.914	1E-08
IFNG	*TBX21*	0.902	0.8	0.953	4E-12
IFNG	*TGFB1*	0.883	0.763	0.944	5E-11
IFNG	*TNFA*	0.871	0.742	0.938	2E-10
IL1B	*IL2*	0.413	0.054	0.677	0.021
IL1B	*IL4*	0.405	0.045	0.672	0.024
IL1B	*IL6*	0.476	0.133	0.717	0.007
IL1B	*LTA*	0.918	0.831	0.961	4E-13
IL1B	*NFKB1*	0.968	0.932	0.985	6E-19
IL1B	*PDCD1*	0.772	0.565	0.887	4E-07
IL1B	*STAT3*	0.968	0.933	0.985	5E-19
IL1B	*STAT5A*	0.967	0.929	0.984	1E-18
IL1B	*TBX21*	0.938	0.872	0.971	6E-15
IL1B	*TGFB1*	0.948	0.891	0.975	6E-16
IL1B	*TNFA*	0.96	0.916	0.981	1E-17
IL2	*IL4*	0.557	0.239	0.767	0.001
IL2	*IL6*	0.59	0.286	0.787	5E-04
IL2	*LTA*	0.529	0.201	0.75	0.002
IL2	*NFKB1*	0.41	0.051	0.675	0.022
IL2	*PDCD1*	0.494	0.156	0.729	0.005
IL2	*STAT3*	0.383	0.02	0.657	0.033
IL2	*STAT5A*	0.356	-0.012	0.639	0.05
IL2	*TBX21*	0.487	0.146	0.724	0.005
IL2	*TGFB1*	0.46	0.112	0.707	0.009
IL2	*TNFA*	0.423	0.067	0.683	0.018

IL4	*IL6*	0.418	0.061	0.68	0.019
IL4	*LTA*	0.447	0.096	0.699	0.012
IL4	*NFKB1*	0.388	0.025	0.66	0.031
IL4	*PDCD1*	0.534	0.208	0.753	0.002
IL4	*STAT3*	0.396	0.034	0.666	0.028
IL4	*STAT5A*	0.365	-0.002	0.645	0.043
IL4	*TBX21*	0.477	0.134	0.718	0.007
IL4	*TGFB1*	0.449	0.098	0.7	0.011
IL4	*TNFA*	0.401	0.04	0.669	0.025
IL6	*LTA*	0.625	0.336	0.807	2E-04
IL6	*NFKB1*	0.509	0.175	0.738	0.003
IL6	*PDCD1*	0.29	-0.086	0.593	0.114
IL6	*STAT3*	0.509	0.176	0.738	0.003
IL6	*STAT5A*	0.471	0.126	0.714	0.007
IL6	*TBX21*	0.668	0.399	0.831	4E-05
IL6	*TGFB1*	0.524	0.195	0.747	0.002
IL6	*TNFA*	0.497	0.16	0.731	0.004
LTA	*NFKB1*	0.932	0.859	0.968	3E-14
LTA	*PDCD1*	0.663	0.391	0.828	5E-05
LTA	*STAT3*	0.925	0.844	0.964	1E-13
LTA	*STAT5A*	0.918	0.831	0.961	4E-13
LTA	*TBX21*	0.935	0.864	0.969	1E-14
LTA	*TGFB1*	0.915	0.825	0.96	6E-13
LTA	*TNFA*	0.924	0.843	0.964	1E-13
NFKB1	*PDCD1*	0.76	0.546	0.881	7E-07
NFKB1	*STAT3*	0.983	0.964	0.992	5E-23
NFKB1	*STAT5A*	0.988	0.975	0.995	3E-25
NFKB1	*TBX21*	0.954	0.902	0.978	1E-16
NFKB1	*TGFB1*	0.984	0.966	0.993	2E-23
NFKB1	*TNFA*	0.974	0.945	0.988	2E-20
PDCD1	*STAT3*	0.753	0.533	0.877	1E-06
PDCD1	*STAT5A*	0.749	0.527	0.875	1E-06
PDCD1	*TBX21*	0.772	0.565	0.887	4E-07
PDCD1	*TGFB1*	0.792	0.599	0.898	1E-07
PDCD1	*TNFA*	0.805	0.623	0.905	5E-08
STAT3	*STAT5A*	0.987	0.972	0.994	1E-24
STAT3	*TBX21*	0.948	0.891	0.975	6E-16
STAT3	*TGFB1*	0.969	0.935	0.986	3E-19
STAT3	*TNFA*	0.97	0.936	0.986	2E-19
STAT5A	*TBX21*	0.943	0.88	0.973	2E-15
STAT5A	*TGFB1*	0.977	0.951	0.989	6E-21
STAT5A	*TNFA*	0.978	0.954	0.99	2E-21
TBX21	*TGFB1*	0.962	0.919	0.982	8E-18
TBX21	*TNFA*	0.962	0.921	0.982	6E-18
TGFB1	*TNFA*	0.974	0.945	0.988	3E-20
