# Supplementary material for: Expression of Sex Hormone Receptor and Immune Response Genes in Peripheral Blood Mononuclear Cells During the Menstrual Cycle
Source: Front Endocrinol (Lausanne). 2021 Sep 22;12:721813. doi: 10.3389/fendo.2021.721813 (PMC8493253; doi:10.3389/fendo.2021.721813)
Supplement: Supplementary file 8 [file DataSheet_8.pdf]

Supplemental Table 4. Gene-hormone correlation.
Repeated measures correlation between PBMC gene expression (-ΔCT) and serum sex hormone (SH) levels in pre-MP women (*n* = 10, each sampled 4 times, df = 27). Genes with no registered expression were omitted (*PGR*, *IL17* and *CYP19A1*). Abbreviations: E_2, Estradiol; LH, Luteinizing hormone; P_4, Progesterone; FSH, Follicle-stimulating hormone; SHBG, Sex hormone-binding globulin. With α set to 0.00032 there was no correlation between genes with any hormone studied, with the exception of SHBG and *ESR2_ERb1* (0.000215).

Gene	SH	r	Lower CI	Upper CI	*p*-value	Gene	SH	r	Lower CI	Upper CI	*p*-value
IL4	E_2	0.316	-0.612	0.057	0.0829	*ESR2_ERb1*	FSH	-0.332	-0.039	0.623	0.0677
NFKB1	E_2	0.295	-0.597	0.081	0.108	*NFKB1*	FSH	-0.141	-0.238	0.483	0.449
LTA	E_2	0.294	-0.596	0.081	0.109	*ESR2_ERb2*	FSH	-0.141	-0.238	0.482	0.450
IFNG	E_2	0.290	-0.594	0.085	0.113	*TGFB1*	FSH	-0.133	-0.245	0.477	0.474
TGFB1	E_2	0.272	-0.580	0.105	0.139	*GATA3*	FSH	-0.125	-0.253	0.470	0.503
ESR2_ERb2	E_2	0.265	-0.576	0.112	0.150	*ESR1_ERa*	FSH	-0.118	-0.260	0.464	0.527
STAT5A	E_2	0.264	-0.575	0.113	0.151	*LTA*	FSH	-0.112	-0.266	0.459	0.550
IL1B	E_2	0.249	-0.564	0.129	0.176	*STAT5A*	FSH	-0.109	-0.268	0.457	0.560
STAT3	E_2	0.242	-0.559	0.137	0.190	*TBX21*	FSH	-0.102	-0.275	0.452	0.584
ESR2_ERb1	E_2	0.232	-0.551	0.147	0.210	*STAT3*	FSH	-0.093	-0.283	0.445	0.617
TNF	E_2	0.229	-0.550	0.150	0.215	*IFNG*	FSH	-0.090	-0.286	0.442	0.632
GATA3	E_2	0.214	-0.538	0.166	0.249	*TNF*	FSH	-0.078	-0.297	0.433	0.675
AR	E_2	0.210	-0.535	0.170	0.257	*IL6*	FSH	-0.062	-0.312	0.419	0.740
PDCD1	E_2	0.172	-0.506	0.208	0.355	*IL4*	FSH	-0.051	-0.321	0.410	0.784
TBX21	E_2	0.152	-0.491	0.227	0.413	*IL1B*	FSH	-0.049	-0.323	0.408	0.792
IL2	E_2	0.128	-0.472	0.250	0.493	*PDCD1*	FSH	-0.020	-0.349	0.384	0.915
ESR1_ERa	E_2	0.097	-0.448	0.279	0.603	*AR*	FSH	0.015	-0.380	0.353	0.935
IL6	E_2	-0.078	-0.297	0.432	0.675	*IL2*	FSH	-0.009	-0.359	0.374	0.961
AR	LH	0.266	-0.576	0.111	0.148	*ESR2_ERb1*	SHBG	0.617	-0.802	-0.324	0.000215
IL4	LH	0.266	-0.576	0.112	0.149	*PDCD1*	SHBG	0.333	-0.623	0.038	0.0671
IL2	LH	0.254	-0.568	0.124	0.168	*TGFB1*	SHBG	0.209	-0.535	0.170	0.259
IFNG	LH	0.241	-0.558	0.138	0.191	*AR*	SHBG	0.194	-0.523	0.186	0.295
LTA	LH	0.224	-0.546	0.155	0.225	*IL6*	SHBG	-0.187	-0.192	0.518	0.313

IL1B	LH	0.224	-0.546	0.155	0.225	*IL4*	SHBG	0.185	-0.516	0.195	0.319
TNF	LH	0.204	-0.530	0.176	0.272	*GATA3*	SHBG	0.182	-0.514	0.198	0.327
TGFB1	LH	0.187	-0.518	0.192	0.313	*TNF*	SHBG	0.179	-0.512	0.200	0.334
ESR2_ERb2	LH	0.180	-0.513	0.200	0.333	*NFKB1*	SHBG	0.179	-0.512	0.200	0.334
STAT5A	LH	0.176	-0.510	0.204	0.344	*STAT5A*	SHBG	0.154	-0.492	0.226	0.410
NFKB1	LH	0.176	-0.509	0.204	0.345	*LTA*	SHBG	0.153	-0.492	0.226	0.412
PDCD1	LH	0.175	-0.509	0.205	0.347	*STAT3*	SHBG	0.152	-0.491	0.227	0.414
STAT3	LH	0.171	-0.506	0.209	0.358	*TBX21*	SHBG	0.152	-0.491	0.227	0.414
TBX21	LH	0.166	-0.502	0.214	0.373	*IL1B*	SHBG	0.146	-0.487	0.233	0.432
GATA3	LH	0.146	-0.487	0.233	0.432	*IFNG*	SHBG	0.143	-0.484	0.236	0.442
ESR1_ERa	LH	0.134	-0.477	0.244	0.472	*ESR1_ERa*	SHBG	0.103	-0.453	0.273	0.580
IL6	LH	0.106	-0.455	0.271	0.572	*ESR2_ERb2*	SHBG	0.071	-0.427	0.303	0.704
ESR2_ERb1	LH	-0.031	-0.339	0.393	0.869	*IL2*	SHBG	0.064	-0.421	0.310	0.733
NFKB1	P_4	0.509	-0.738	-0.176	0.00342	*PDCD1*	T	0.324	-0.617	0.048	0.0751
LTA	P_4	0.504	-0.735	-0.169	0.00381	*IL6*	T	-0.161	-0.218	0.498	0.386
STAT5A	P_4	0.474	-0.716	-0.131	0.00700	*IL1B*	T	0.153	-0.492	0.226	0.412
TGFB1	P_4	0.474	-0.716	-0.130	0.00708	*IFNG*	T	0.152	-0.491	0.228	0.416
STAT3	P_4	0.472	-0.715	-0.127	0.00737	*TNF*	T	0.118	-0.464	0.260	0.529
GATA3	P_4	0.457	-0.705	-0.109	0.00978	*AR*	T	0.113	-0.461	0.264	0.544
IL1B	P_4	0.434	-0.691	-0.080	0.0147	*STAT3*	T	0.091	-0.443	0.285	0.627
TNF	P_4	0.426	-0.685	-0.070	0.0169	*NFKB1*	T	0.087	-0.440	0.288	0.640
TBX21	P_4	0.400	-0.669	-0.040	0.0257	*ESR2_ERb1*	T	-0.077	-0.298	0.432	0.679
IFNG	P_4	0.387	-0.660	-0.024	0.0315	*STAT5A*	T	0.077	-0.431	0.298	0.681
PDCD1	P_4	0.296	-0.597	0.080	0.106	*ESR1_ERa*	T	-0.074	-0.300	0.429	0.691
IL4	P_4	0.240	-0.557	0.139	0.194	*IL4*	T	0.066	-0.422	0.308	0.725
ESR1_ERa	P_4	0.227	-0.548	0.152	0.218	*GATA3*	T	0.043	-0.403	0.328	0.817
ESR2_ERb1	P_4	0.225	-0.547	0.154	0.223	*TGFB1*	T	0.043	-0.403	0.328	0.817
IL6	P_4	0.080	-0.434	0.295	0.670	*TBX21*	T	0.038	-0.399	0.333	0.838
IL2	P_4	0.063	-0.420	0.311	0.737	*LTA*	T	-0.027	-0.343	0.389	0.887
ESR2_ERb2	P_4	-0.025	-0.344	0.388	0.892	*IL2*	T	0.011	-0.376	0.357	0.955

AR	P_4	-0.015	-0.354	0.379	0.938	*ESR2_ERb2*	T	0.003	-0.369	0.364	0.987
